# Intrathyroidal Hematoma, Biphasic Thyroid Dysfunction, and Permanent Hypothyroidism After Fine Needle Aspiration Biopsy

**DOI:** 10.1016/j.aed.2026.04.002

**Published:** 2026-04-20

**Authors:** Mohammad Jay, Rebecca B.K. Wong, Cristina Goens, Jeannette Goguen

**Affiliations:** 1 Division of Endocrinology, Department of Medicine, University of Toronto, Toronto, Ontario, Canada; 2 Department of Medicine, Western University, London, Ontario, Canada

**Keywords:** destructive thyroiditis, fine-needle aspiration, hypothyroidism, levothyroxine therapy, thyroid hematoma, thyrotoxicosis

## Abstract

**Background/Objective:**

Fine-needle aspiration (FNA) biopsy is a standard and generally safe procedure for evaluating thyroid nodules. Minor complications such as local pain or small hematomas are relatively common; however, large intrathyroidal hematomas and associated thyroid dysfunction are exceedingly rare.

**Case Report:**

We report the case of a 37-year-old woman who developed acute neck pain and swelling 1 week after FNA of a right thyroid nodule. Imaging revealed a large intrathyroidal hematoma extending across the isthmus. Thyroid function testing showed transient thyrotoxicosis followed by severe hypothyroidism, requiring long-term levothyroxine replacement. The patient had no history of bleeding disorders, anticoagulant or antiplatelet use, and experienced no procedural difficulties at the time of biopsy.

**Discussion:**

This case illustrates a rare but clinically important complication of FNA, in which mechanical and inflammatory injury from a hematoma led to destructive thyroiditis and permanent thyroid failure.

**Conclusion:**

Awareness of this potential complication, even among low-risk individuals, highlights the importance of avoiding FNA if not indicated, counseling patients on postprocedure symptoms and monitoring thyroid function in those who develop pain or swelling after biopsy.

**Highlights**

• Fine needle aspiration of thyroid nodules can rarely cause permanent thyroid injury
• Large intrathyroidal hematomas may lead to destructive thyroiditis
• Thyroid trauma can cause transient hyperthyroidism followed by hypothyroidism
• Delayed neck pain or swelling after biopsy warrants urgent evaluation
• Long-term thyroid hormone replacement may be required after biopsy-related injury

**Clinical Relevance**

Although thyroid fine-needle aspiration biopsy is generally safe, rare complications can cause clinically important endocrine injury. This case emphasizes the need for careful patient counseling, recognition of delayed symptoms, and timely evaluation to reduce morbidity and enable early detection of thyroid dysfunction.

## Introduction

Thyroid nodules are common, with ultrasound studies reporting a prevalence of 19% to 68% in the general population.^1^, ^2^, ^3^ Fine-needle aspiration (FNA) is the standard diagnostic procedure for evaluating thyroid with suspicious sonographic features. FNA is generally safe, with most complications limited to mild local pain or small self-limited hematomas.^4^

Serious adverse events are rare but have been reported, including large intrathyroidal hematomas, airway compromise, and thyroid dysfunction.^5^, ^6^, ^7^ We describe a rare case of an extensive intrathyroidal hematoma developing 1 week after FNA, followed by biphasic thyroid dysfunction characterized by transient thyrotoxicosis and subsequent prolonged hypothyroidism, consistent with trauma-induced destructive thyroiditis. This case highlights the importance of recognizing FNA-related thyroid injury as a potential, although uncommon, cause of permanent glandular dysfunction.

## Case Presentation

A 37-year-old woman with a history of macroprolactinoma, well controlled on cabergoline 0.5 mg weekly with normal prolactin levels, was referred for evaluation of a right thyroid nodule. There were no clinical or biochemical features suggestive of pituitary dysfunction or central hypothyroidism. She had no known thyroid disease and was clinically and biochemically euthyroid prior to biopsy. Thyroid function testing was performed as part of the initial clinical evaluation and not as a procedural prerequisite for FNA. Thyroid peroxidase and thyroglobulin antibodies were negative.

She denied compressive symptoms and had no personal or family history of bleeding disorders or thyroid malignancy. She was not taking anticoagulants, antiplatelet agents, or other medications known to affect coagulation. Prebiopsy laboratory testing showed normal hemoglobin 113 g/L (11.3 g/dL; reference 115–155 g/L [11.5–15.5 g/dL]) and platelet count 170 × 10^9^/L (170 × 10^3^/μL; reference 140–400 × 10^9^/L [140–400 × 10^3^/μL]). Formal coagulation parameters were not available. There was no history of abnormal bleeding with previous procedures.

**Fig. 1:**
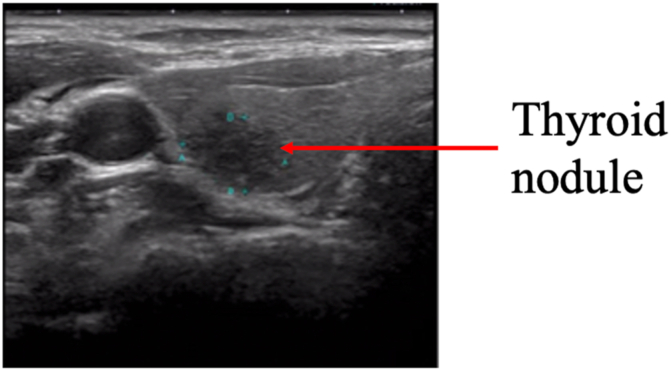
Ultrasound image of the *right* thyroid lobe obtained in 2016, demonstrating a hypoechoic nodule in the *lower* pole (1.3 × 0.9 × 0.6 cm) prior to fine-needle aspiration.

Neck ultrasound on May 8, 2017 revealed a 1.3 × 0.9 × 0.6 cm hypoechoic nodule in the lower pole of the right thyroid lobe, showing less than 20% growth compared with prior imaging (Fig. 1). According to the 2015 American Thyroid Association (ATA) Management Guidelines for Adult Patients with Thyroid Nodules and Differentiated Thyroid Cancer, the nodule was classified as intermediate suspicion (estimated malignancy risk 10% to 20%).^8^ Retrospectively, these features corresponded to the American College of Radiology (ACR) TI-RADS category 4 (moderate suspicion; estimated malignancy risk approximately 5% to 20%).^9^

An ultrasound-guided FNA was performed on May 18, 2017 in accordance with the 2015 ATA recommendations.^8^ The procedure was well tolerated with no immediate complications, and cytology was nondiagnostic (Bethesda I). One week later, the patient developed acute right-sided neck pain and swelling. She was afebrile and hemodynamically stable. No stridor or hoarseness was observed, and she reported no dysphagia. Further imaging was performed to evaluate for postprocedural complications.

**Fig. 2:**
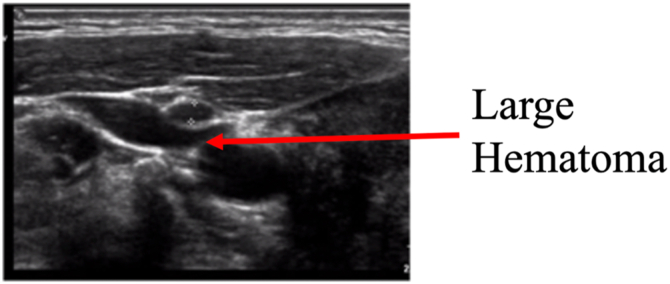
Ultrasound image obtained 2 weeks after fine-needle aspiration in 2017, showing a heterogeneous hypoechoic area (3.3 × 2.2 × 1.3 cm) within the *right* thyroid lobe, consistent with an evolving intrathyroidal hematoma.

Ultrasound on June 27, 2017 (5 weeks after FNA) demonstrated a new, ill-defined, heterogeneous hypoechoic area measuring 3.3 × 2.2 × 1.3 cm within the right thyroid lobe, extending across the isthmus into the left lobe (Fig. 2). The appearance was interpreted as an evolving intrathyroidal hematoma. No drainable fluid collection, cervical lymphadenopathy, or parathyroid abnormality was identified. Inflammatory markers were not obtained.

A repeat ultrasound on July 31, 2017 showed interval enlargement of the hypoechoic region, now involving nearly the entire right lobe and extending further across the isthmus. The radiologist noted that the appearance was atypical for post-FNA changes, and an evolving hematoma remained the leading consideration.

Serial follow-up ultrasounds demonstrated gradual resolution. By August 28, the hematoma had decreased in size, and by September 28, it measured 1.2 × 0.7 cm. On December 3, 2017, complete resolution was observed, with restoration of a homogeneous parenchymal texture. The originally biopsied nodule was no longer visible.

Thyroid function testing 1 month after FNA revealed a suppressed thyroid stimulating hormone (TSH) level <0.01 mU/L with a normal free T4, consistent with subclinical thyrotoxicosis. Free T3 was slightly elevated at 6.1 pmol/L (reference 3.8–6.0 pmol/L), and thyrotropin receptor antibodies were not measured. There were no clinical features of Graves’ disease, such as ophthalmopathy, thyroid bruit, or pretibial myxedema. The pattern was consistent with trauma-induced thyroiditis.

Three months after FNA, thyroid function showed severe hypothyroidism, with TSH 285.5 mU/L and free T4 < 2.0 pmol/L. Antithyroid peroxidase and antithyroglobulin antibodies were negative. Clinically, she reported alternating heat and cold intolerance but no weight change or galactorrhea. Menses were regular. Examination revealed a nontender thyroid without palpable nodules. Deep tendon reflexes, skin texture, and extraocular movements were normal, and visual fields were full to confrontation.

The patient’s hematoma was managed conservatively, as there was no evidence of airway compromise, hemodynamic instability, or active bleeding. Similarly, no treatment was administered for her subclinical thyrotoxicosis. She was closely monitored with serial thyroid ultrasounds and thyroid function testing.

Levothyroxine 50 mcg daily was initiated in October 2017 for overt hypothyroidism. Despite treatment, TSH remained elevated at 95.4 mU/L in December 2017, prompting dose escalation to 75 mcg and subsequently to 100 mcg daily. Over the following year, thyroid function tests normalized and symptoms resolved. The levothyroxine dose was later reduced and stabilized at 88 mcg daily, maintaining biochemical euthyroidism.

**Fig. 3:**
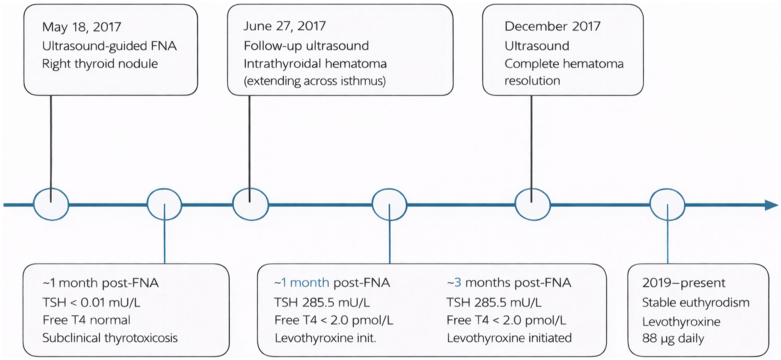
Clinical timeline following fine-needle aspiration complicated by intrathyroidal hematoma and biphasic thyroid dysfunction. Timeline depicting the temporal relationship between fine-needle aspiration (FNA), symptom onset, imaging evolution of the intrathyroidal hematoma, thyroid function changes (transient thyrotoxicosis followed by severe hypothyroidism), and initiation of levothyroxine therapy. TSH, thyroid stimulating hormone.

Follow-up ultrasound in December 2017 confirmed complete resolution of the hematoma and involution of the previously biopsied nodule. No new nodules or masses were identified on subsequent imaging. The most recent thyroid ultrasound in January 2020 showed no recurrence of hematoma or new thyroid abnormalities. Overall, the clinical course followed a clear temporal sequence: acute neck symptoms developed approximately 1 week after FNA; ultrasound at 5 weeks demonstrated an evolving intrathyroidal hematoma; thyroid function testing at 1 month showed transient thyrotoxicosis; and by 3 months the patient had developed overt hypothyroidism requiring levothyroxine (Fig. 3).

Over a 7-year follow-up period, the patient remained clinically euthyroid and asymptomatic on levothyroxine 88 mcg daily, with TSH levels ranging from 0.5 to 2.3 mU/L between 2019 and 2024. Serial thyroid function results and corresponding levothyroxine doses throughout the clinical course are summarized in Table 1.

**Table 1:** Serial Thyroid Function Tests (TSH and Free T4) and Corresponding Levothyroxine Dosages Over Time, With Most Recent Results at the Top

Date (DD/MM/YY)	TSH (mU/L)	Free T4 (pmol/L)	Levothyroxine dose (mcg daily)
23/05/24	0.52 (0.52)	10.9 (0.85)	88
30/05/22	1.87 (1.87)	9.6 (0.75)	88
13/09/21	9.34 (9.34)	10.5 (0.82)	88
13/04/21	0.34 (0.34)	13.9 (1.08)	88
10/01/20	7.48 (7.48)	9.5 (0.74)	100
28/03/19	2.30 (2.30)	—	100
18/10/18	5.75 (5.75)	7.2 (0.56)	100
20/04/18	30.62 (30.62)	<2.0 (<0.16)	75
08/12/17	95.36 (95.36)	—	50
08/10/17	285.48 (285.48)	—	50
09/08/17	<0.01 (<0.01)	—	None
14/06/17	2.17 (2.17)	—	None
27/05/16	5.52 (5.52)	—	None
24/01/14	5.87 (5.87)	—	None

Abbreviations: TSH, thyroid-stimulating hormone; T4, thyroxine.

Reference ranges: TSH 0.4–5.5 mU/L Free T4 7.5–16.0 pmol/L.

## Discussion

This case illustrates a rare but clinically significant complication of FNA. The patient developed a large intrathyroidal hematoma followed by biphasic thyroid dysfunction, with transient thyrotoxicosis and subsequent prolonged hypothyroidism. This pattern is most consistent with trauma-induced destructive thyroiditis. The initial thyrotoxicosis likely resulted from passive release of preformed thyroid hormones due to follicular disruption, whereas the later hypothyroid phase reflected irreversible follicular cell loss.

Most FNA complications are minor, limited to local discomfort or small, self-limited hematomas.^4^ Large hematomas are uncommon, with an estimated incidence below 1%, and are rarely associated with airway compromise or permanent thyroid dysfunction.^7^^,^^10^ A systematic review found no difference in bleeding risk between patients taking and not taking anticoagulant therapy, supporting classification of thyroid FNA as a low–bleeding-risk procedure.^10^^,^^11^ Fatal post-FNA hemorrhages, although exceedingly rare, have nevertheless been reported.^12^

The delayed onset of hematoma in this case, occurring 1 week after FNA, is unusual, as most hemorrhagic complications develop within 24 h.^13^ Proposed mechanisms include fragile intrathyroidal vasculature or unrecognized arteriovenous shunts that may predispose to delayed venous bleeding.^14^^,^^15^ The absence of anticoagulant exposure or coagulopathy emphasizes the unpredictable nature of this complication.

Although the intrathyroidal hematoma did not involve the entire gland and subsequently resolved radiologically, persistent hypothyroidism may reflect irreversible loss of functional thyroid parenchyma. Autoimmune thyroid disease is unlikely, as antithyroid peroxidase and antithyroglobulin antibodies were negative both prior to biopsy and at the time of overt hypothyroidism 3 months later. Partial destruction of thyroid tissue, even when localized, can exceed the compensatory capacity of the remaining gland, analogous to the development of hypothyroidism following hemithyroidectomy. In this context, localized parenchymal injury related to the hematoma likely contributed to permanent thyroid dysfunction.

Thyroid dysfunction after FNA is rare. A 1992 case series by Kobayashi et al described transient thyrotoxicosis occurring 2–20 days after FNA, resolving spontaneously within 1 month.^16^ Subsequent reports have documented isolated cases of post-FNA thyrotoxicosis, thyroiditis, or acute suppurative thyroiditis, most resolving without long-term sequelae (Table 2).^5^^,^^6^^,^^17^^,^^18^ Lamos and Munir reported the only previously published case demonstrating a biphasic pattern of transient hyperthyroidism followed by hypothyroidism, though without hematoma formation and in the context of exogenous human chorionic gonadotropin use.^6^ To our knowledge, our case is the first reported instance of hematoma-associated destructive thyroiditis leading to permanent hypothyroidism.

**Table 2:** Published Reports Describing Thyroid Dysfunction or Related Complications Following Fine-Needle Aspiration (FNA) Biopsy

Report	Case details	Time from FNA to dysfunction (d)	Outcome
Kobayashi et al, 1992^16^	Retrospective analysis: 5 of 500 patients developed transient thyrotoxicosis after FNA of thyroid cysts. (resolved within 2–4 wk).	2–20	Recovered spontaneously in ≤1 mo
Nishihara et al, 2005^17^	39 y female; Staphylococcus aureus infection after third FNA of left thyroid cyst → thyrotoxicosis; treated with antibiotics.	4	Full recovery 1 mo
Orrego, 2016^18^	78 y female with spongiform nodule; thyrotoxicosis 15 d post-FNA; treated with methimazole.	15	Euthyroid within 5 mo
Lamos & Munir, 2016^6^	60 y female with multinodular goiter on hCG injections; developed thyroiditis then hypothyroidism requiring levothyroxine.	≈30	Permanent hypothyroidism
Eatz et al, 2023^5^	62 y female with Graves’ disease; thyroid storm 1 d post-FNA → intubation and thyroidectomy.	1	Recovered postthyroidectomy
*Present case*	37 y female with no coagulopathy; large hematoma 1 wk after FNA → biphasic thyroid dysfunction and permanent hypothyroidism.	≈7	Permanent hypothyroidism

Abbreviations: FNA, fine-needle aspiration; hCG, human chorionic gonadotropin.

Early recognition of post-FNA complications is crucial. Patients should be advised to seek urgent medical attention for progressive neck pain, swelling, or dyspnea, which may indicate hematoma formation, and for symptoms suggestive of thyroid dysfunction. Conservative management of hematoma is appropriate when there is no airway compromise, with close follow-up using serial imaging and thyroid function monitoring as indicated. Thyroid function testing should be performed if biochemical or clinical features of thyrotoxicosis or hypothyroidism develop. In retrospect, earlier thyroid function assessment in our patient might have identified evolving hypothyroidism sooner, highlighting the importance of follow-up testing when post-FNA symptoms occur. Repeat ultrasonography with Doppler may help identify vascular abnormalities in higher-risk patients or those with atypical anatomy.

The indication for biopsy in this case reflected standard practice at the time, consistent with the 2015 ATA guidelines recommending FNA for intermediate-suspicion nodules ≥1 cm.^8^ Subsequent evidence suggests that ACR TI-RADS maintains malignancy detection while recommending fewer biopsies, thereby reducing exposure to procedure-related risks.^9^^,^^19^ This evolution in risk-stratification frameworks highlights how refinements in guideline-based decision-making can enhance patient safety.

While FNA remains the gold standard diagnostic procedure and is overwhelmingly safe, this case highlights the importance of awareness of rare complications with meaningful endocrine sequelae. Careful patient counseling and prompt evaluation of postprocedural symptoms can help reduce morbidity and facilitate early recognition of FNA-related thyroid injury.

## Patient Consent

The authors have obtained written patient consent for use of the images.

## Disclosure

The authors have no conflicts of interest to disclose.
